# GRSR: a tool for deriving genome rearrangement scenarios from multiple unichromosomal genome sequences

**DOI:** 10.1186/s12859-018-2268-1

**Published:** 2018-08-13

**Authors:** Dan Wang, Lusheng Wang

**Affiliations:** 1 Department of Computer Science, City University of Hong Kong, 83 Tat Chee Ave., Hong Kong, People’s Republic of China; 2 University of Hong Kong Shenzhen Research Institute, Shenzhen Hi-Tech Industrial Park, Nanshan District, Shenzhen, People’s Republic of China

**Keywords:** Comparative genomics, Genome rearrangement, Reversal, Transposition, Block interchange

## Abstract

**Background:**

Genome rearrangements describe changes in the genetic linkage relationship of large chromosomal regions, involving reversals, transpositions, block interchanges, deletions, insertions, fissions, fusions and translocations *etc*. Many algorithms for calculating rearrangement scenarios between two genomes have been proposed. Very often, the calculated rearrangement scenario is not unique for the same pair of permutations. Hence, how to decide which calculated rearrangement scenario is more biologically meaningful becomes an essential task. Up to now, several mechanisms for genome rearrangements have been studied. One important theory is that genome rearrangement may be mediated by repeats, especially for reversal events. Many reversal regions are found to be flanked by a pair of inverted repeats. As a result, whether there are repeats at the breakpoints of the calculated rearrangement events can shed a light on deciding whether the calculated rearrangement events is biologically meaningful. To our knowledge, there is no tool which can automatically identify rearrangement events and check whether there exist repeats at the breakpoints of each calculated rearrangement event.

**Results:**

In this paper, we describe a new tool named GRSR which allows us to compare multiple unichromosomal genomes to identify “independent” (obvious) rearrangement events such as reversals, (inverted) block interchanges and (inverted) transpositions and automatically searches for repeats at the breakpoints of each rearrangement event. We apply our tool on the complete genomes of 28 *Mycobacterium tuberculosis* strains and 24 *Shewanella* strains respectively. In both *Mycobacterium tuberculosis* and *Shewanella* strains, our tool finds many reversal regions flanked by a pair of inverted repeats. In particular, the GRSR tool also finds an inverted transposition and an inverted block interchange in *Shewanella*, where the repeats at the ends of rearrangement regions remain unchanged after the rearrangement event. To our knowledge, this is the first time such a phenomenon for inverted transposition and inverted block interchange is reported in *Shewanella*.

**Conclusions:**

From the calculated results, there are many examples supporting the theory that the existence of repeats at the breakpoints of a rearrangement event can make the sequences at the breakpoints remain unchanged before and after the rearrangement events, suggesting that the conservation of ends could possibly be a popular phenomenon in many types of genome rearrangement events.

## Background

Genome rearrangements involve gross changes of chromosomes and play important role in speciation. The problem of sorting signed genomic permutations arises in the study of species evolution via genome rearrangement. In this problem, chromosomes of interest are denoted by permutations of signed and ordered integers with each integer represents a conserved region (synteny block) in chromosomes. The order of the integers describes the order of synteny blocks on the chromosome and the sign (+ or −) of each integer indicates the transcriptional orientation. In the problem of sorting signed genomic permutations, given a pair of permutations and a set of rearrangement operations, we need to compute the minimum number of rearrangement operations in the set required to transform one permutation into another. The set of rearrangement operations can include one or more kinds of rearrangement operations, such as reversal, transposition, block interchange, fusion, fission, etc.

Many tools or algorithms have been proposed for sorting signed genomic permutations. In 1995, Hannenhalli and Pevzner provided a polynomial time algorithm for computing the minimum number of reversals, translocations, fissions, and fusions that would transform one multi-chromosomal genome to another [1]. Bafna and Pevzner proposed an algorithm for sorting permutations by transposition [2]. In 2002, Tesler improved the algorithm of Hannenhalli and Pevzner and proposed an efficient algorithm for multichromosomal genome rearrangements [3] and developed a tool named GRIMM to implement his algorithm [4]. SPRING is a tool for the analysis of genome rearrangement using reversals and block-interchanges [5].

In fact, given a pair of permutations, there are often more than one optimal rearrangement scenarios, especially when the rearrangement distance between this permutation pair is large. And sometimes, for the same pair of permutations, the computed rearrangement scenarios using different tools are not consistent. Hence, how to know whether the calculated scenarios are solid and biologically meaningful becomes an essential task.

Up to now, several mechanisms for genome rearrangements have been reported [6, 7]. Statistics analyzes showed that breakpoints are often associated with repetitive elements [8, 9]. There was evidence showing that a reversal can be mediated by a pair of inverted repeats (IRs) [10]. Rajaraman et al. suggested that rearrangements could be driven by the Insertion Sequences (ISs) and the positions of the reversal breakpoints in their study were also highly correlated with IS [11]. Darmon and Leach reviewed many examples of prokaryotic genomic rearrangements which were induced by natural transposable elements and pointed out that recombination between IRs can result in a reversal of the internal DNA sequence [6]. The association between IR and genome rearrangement breakpoints was also reported in previous studies on mammals and drosophila genomes [12–14]. Hence, whether there exist repeats at the breakpoints of rearrangement events may give us a clue on whether the calculated rearrangement scenarios are biologically meaningful. To our knowledge, there is no tool which can automatically identify rearrangement events and check whether there exist repeats at the breakpoints of each calculated rearrangement event.

In this paper, we describe a new tool named GRSR for deriving genome rearrangement scenarios from multiple unichromosomal genome sequences and checking whether there are repeats at the breakpoints of each calculated rearrangement event. The input of the GRSR tool is a set of unichromosomal genome sequences and the output is pairwise rearrangement scenario which is a series of transpositions, block interchanges and reversals. Besides, for each calculated rearrangement event, GRSR checks whether there exist repeats which may mediate this rearrangement event. We applied the GRSR tool on complete genomes of 28 *Mycobacterium tuberculosis* strains and 24 *Shewanella* strains, respectively. We found many examples for supporting the theory that the existence of repeats at the breakpoints of the rearrangement can make the sequences at the breakpoints remain unchanged before and after the rearrangement events.

## Methods

The GRSR tool can derive the pairwise rearrangement scenario and find repeats which may mediate a genome rearrangement event. The GRSR tool is comprised of four primary steps:

1. a multiple sequence alignment of the input genomes using Mugsy [15];
2. extraction of the coordinates of core blocks (sequences shared by all of the input genomes);
3. construction of synteny blocks and generating a signed permutation for each of the input genomes using GRIMM [4];
4. calculation of pairwise rearrangement scenarios using GRSR specific codes and GRIMM [4] and identification of repeats which may mediate rearrangement events using BLAST [16].

The input of the GRSR tool consists of multiple unichromosomal genomes (one file per genome). GRSR provides a Shell wrapper script for each of the four primary steps. Firstly, we use Mugsy to conduct a multiple sequence alignment of the input genomes and the alignment result is in an MAF file. Secondly, as transpositions, block interchanges and reversals happen on sequences which are shared by genomes, we extract the coordinates of core blocks (shared by all of the input genomes) from the MAF file. Thirdly, we utilize the coordinates of core blocks to construct synteny blocks using GRIMM [4] and each input genome will be represented by a signed permutation describing the synteny block order on its chromosome. Lastly, we implement a novel method to compute the pairwise rearrangement scenario which is a series of rearrangement events involved in transforming one genome’s permutation into another. The computed rearrangement scenarios will only include rearrangement events which happen on a single chromosome, such as transposition, block interchange and reversals. Once getting a rearrangement event, the GRSR tool will check whether there are repeats at the breakpoints of this event. The GRSR tool writes the rearrangement scenarios and whether there are repeats at the breakpoints of each rearrangement event into the *report.txt* file.

### Multiple sequence alignment

We use Mugsy to conduct multiple sequence alignment of the input genomes. The alignment result is written to a file in the MAF format (See the “Availability of data and materials” section). In the MAF file, there are *core blocks* which include sequences shared by all of the input genomes and *accessory blocks* which include sequences shared by a subset of the input genomes.

### Extraction of the coordinates of core blocks

As transposition, block interchange and reversals happen on sequences shared by genomes, we write a Shell script to extract the coordinates of core blocks on the input genomes. The coordinates of core blocks are written into a file named *core_coords.txt* (See the “Availability of data and materials” section) in the format as required by GRIMM.

### Construction of synteny blocks and generating signed permutations

By utilizing the coordinates of core blocks, the GRIMM-SYNTENY tool inside the GRIMM package can construct synteny blocks and generate signed permutations for the input genomes. The coordinates of the synteny blocks on the input genomes are written into a file named *blocks.txt* and the signed permutations for the input genomes are saved in a file named *mgr_macro.txt* (See the “Availability of data and materials” section).

### Calculation of pairwise rearrangement scenarios and finding repeats at the breakpoints of rearrangement events

For every pair of the input genomes, the GRSR tool will produce a rearrangement scenario. Hence, for n input genomes, the GRSR will produce rearrangement scenarios for $C_{n}^{2}$ pairs of genomes.

Given a pair of signed permutations *s* and *d*, the GRSR tool calculate rearrangement scenario from *s* to *d* in three steps:

**Step 1: merging blocks which are in the same order on*s* and*d*.** For example, if

1 $$\begin{array}{*{20}l} s &= 1, 2, 3, 4, 5, 6, 7, 8,9 \\ d &= 1, 2, 3, 7, -6, -5, -4, 8,-9 \end{array} $$

after merging blocks,

2 $$\begin{array}{*{20}l} &s = 1, 4, 7, 8,9 \\ &d = 1, 7, -4, 8,-9 \end{array} $$

In the above example, as Block 1, 2 and 3 in Eq. 1 are in the same order on *s* and *d*, GRSR uses Block 1 in Eq. 2 to replace Block 1, 2 and 3 in Eq. 1. For the same reason, as Block 4, 5 and 6 in Eq. 1 are in the same order on *s* and *d*, the GRSR tool uses Block 4 in Eq. 2 to replace Block 4, 5 and 6 in Eq. 1 on *s* and use Block -4 in Eq. 2 to replace Block -6, -5 and -4 in Eq. 1 on *d*. We can find that merging blocks which are in the same order on *s* and *d* will not change the rearrangement scenarios from *s* to *d*.

**Step 2: detecting independent transposition and block interchange.** In a rearrangement scenario between two permutations, blocks which are involved in only one transposition or block interchange event are defined as blocks which are involved in an *independent* transposition or block interchange. After merging blocks in Step 1, the GRSR tool detects and removes blocks which are involved in *independent* transpositions or block interchanges before computing the reversal events using GRIMM.

Here we show all the cases of *independent* transpositions and block interchanges and represent *permutation s* as:

3 $$ s = \ldots,\pi_{i-1}, \pi_{i}, \pi_{i+1}, \ldots, \pi_{j-1}, \pi_{j}, \pi_{j+1}, \ldots  $$

where *π*_*i*_ and *π*_*j*_ are arbitrary synteny blocks at respective position i and j on *permutation s* and i ≠ j and 1 ≤ i, j ≤ n (n is the total number of synteny blocks on *permutation s*). If *π*_*i*_ is *π*_1_, then *π*_*i*−1_ will be *π*_*n*_. If *π*_*i*_ is *π*_*n*_, then *π*_*i*+1_ will be *π*_1_.

We can say *π*_*i*_ is involved in an *independent* transposition from *s* to *d* once we find *π*_*i*_ on *permutation d* such that

4 $$\begin{array}{*{20}l} &d = \ldots, \pi_{i-1}, \pi_{i+1}, \ldots, \pi_{j-1}, \underline{\pi_{i}}, \pi_{j}, \ldots \\ &\text{or} \\ &d = \ldots,\pi_{i-1}, \pi_{i+1}, \ldots, \text{-}\pi_{j}, \underline{\pi_{i}}, \text{-}\pi_{j-1}, \ldots \\ &\text{or}\\ &d = \ldots,\text{-}\pi_{i+1}, \text{-}\pi_{i-1}, \ldots, \pi_{j-1}, \underline{\text{-}\pi_{i}}, \pi_{j}, \ldots \\ &\text{or} \\ &d = \ldots,\text{-}\pi_{i+1}, \text{-}\pi_{i-1}, \ldots, \text{-}\pi_{j}, \underline{\text{-}\pi_{i}}, \text{-}\pi_{j-1}, \ldots  \end{array} $$

We can say *π*_*i*_ is involved in an *independent* inverted transposition from *s* to *d* once we find *π*_*i*_ on *permutation d* such that

5 $$\begin{array}{*{20}l} &d = \ldots, \pi_{i-1}, \pi_{i+1}, \ldots, \pi_{j-1}, \underline{\text{-}\pi_{i}}, \pi_{j}, \ldots \\ &\text{or} \\ &d = \ldots,\pi_{i-1}, \pi_{i+1}, \ldots, \text{-}\pi_{j}, \underline{\text{-}\pi_{i}}, \text{-}\pi_{j-1}, \ldots \\ &\text{or}\\ &d = \ldots,\text{-}\pi_{i+1}, \text{-}\pi_{i-1}, \ldots, \pi_{j-1}, \underline{\pi_{i}}, \pi_{j}, \ldots \\ &\text{or} \\ &d = \ldots,\text{-}\pi_{i+1}, \text{-}\pi_{i-1}, \ldots, \text{-}\pi_{j}, \underline{\pi_{i}}, \text{-}\pi_{j-1}, \ldots  \end{array} $$

We can say *π*_*i*_ and *π*_*j*_ are involved in an *independent* block interchange from *s* to *d* once we find *π*_*i*_ and *π*_*j*_ on *permutation d* such that

6 $$\begin{array}{*{20}l} &d = \ldots, \pi_{i-1}, \underline{\pi_{j}}, \pi_{i+1}, \ldots, \pi_{j-1}, \underline{\pi_{i}}, \pi_{j+1}, \ldots \\ &\text{or} \\ &d = \ldots, \pi_{i-1}, \underline{\text{-}\pi_{j}}, \pi_{i+1}, \ldots, \text{-}\pi_{j+1}, \underline{\pi_{i}}, \text{-}\pi_{j-1}, \ldots \\ &\text{or}\\ &d = \ldots,\text{-} \pi_{i+1}, \underline{\pi_{j}}, \text{-}\pi_{i-1}, \ldots, \pi_{j-1}, \underline{\text{-}\pi_{i}}, \pi_{j+1}, \ldots \\ &\text{or} \\ &d = \ldots,\text{-} \pi_{i+1}, \underline{\text{-}\pi_{j}}, \text{-}\pi_{i-1}, \ldots, \text{-}\pi_{j+1}, \underline{\text{-}\pi_{i}}, \text{-}\pi_{j-1}, \ldots  \end{array} $$

We can say *π*_*i*_ and *π*_*j*_ are involved in an *independent* inverted block interchange from *s* to *d* once we find *π*_*i*_ and *π*_*j*_ on *permutation d* such that

7 $$\begin{array}{*{20}l} &d = \ldots, \pi_{i-1}, \underline{\text{-}\pi_{j}}, \pi_{i+1}, \ldots, \pi_{j-1}, \underline{\text{-}\pi_{i}}, \pi_{j+1}, \ldots \\ &\text{or} \\ &d = \ldots, \pi_{i-1}, \underline{\pi_{j}}, \pi_{i+1}, \ldots, \text{-}\pi_{j+1}, \underline{\text{-}\pi_{i}}, \text{-}\pi_{j-1}, \ldots \\ &\text{or}\\ &d = \ldots,\text{-} \pi_{i+1}, \underline{\text{-}\pi_{j}}, \text{-}\pi_{i-1}, \ldots, \pi_{j-1}, \underline{\pi_{i}}, \pi_{j+1}, \ldots \\ &\text{or} \\ &d = \ldots,\text{-} \pi_{i+1}, \underline{\pi_{j}}, \text{-}\pi_{i-1}, \ldots, \text{-}\pi_{j+1}, \underline{\pi_{i}}, \text{-}\pi_{j-1}, \ldots  \end{array} $$

We can say *π*_*i*_ and *π*_*j*_ are involved in an *independent* half inverted block interchange from *s* to *d* once we find *π*_*i*_ and *π*_*j*_ on *permutation d* such that

8 $$\begin{array}{*{20}l} &d = \ldots, \pi_{i-1}, \underline{\pi_{j}}, \pi_{i+1}, \ldots, \pi_{j-1}, \underline{\text{-}\pi_{i}}, \pi_{j+1}, \ldots \\ &\text{or} \\ &d = \ldots, \pi_{i-1}, \underline{\text{-}\pi_{j}}, \pi_{i+1}, \ldots, \pi_{j-1}, \underline{\pi_{i}}, \pi_{j+1}, \ldots \\ &\text{or} \\ &d = \ldots, \pi_{i-1}, \underline{\pi_{j}}, \pi_{i+1}, \ldots, \text{-}\pi_{j+1}, \underline{\pi_{i}}, \text{-}\pi_{j-1}, \ldots \\ &\text{or}\\ &d = \ldots, \pi_{i-1}, \underline{\text{-}\pi_{j}}, \pi_{i+1}, \ldots, \text{-}\pi_{j+1}, \underline{\text{-}\pi_{i}}, \text{-}\pi_{j-1}, \ldots \\ &\text{or} \\ &d = \ldots,\text{-} \pi_{i+1}, \underline{\text{-}\pi_{j}}, \text{-}\pi_{i-1}, \ldots, \pi_{j-1}, \underline{\text{-}\pi_{i}}, \pi_{j+1}, \ldots \\ &\text{or} \\ &d = \ldots,\text{-} \pi_{i+1}, \underline{\pi_{j}}, \text{-}\pi_{i-1}, \ldots, \pi_{j-1}, \underline{\pi_{i}}, \pi_{j+1}, \ldots \\ &\text{or} \\ &d = \ldots,\text{-} \pi_{i+1}, \underline{\pi_{j}}, \text{-}\pi_{i-1}, \ldots, \text{-}\pi_{j+1}, \underline{\text{-}\pi_{i}}, \text{-}\pi_{j-1}, \ldots \\ &\text{or} \\ &d = \ldots,\text{-} \pi_{i+1}, \underline{\text{-}\pi_{j}}, \text{-}\pi_{i-1}, \ldots, \text{-}\pi_{j+1}, \underline{\pi_{i}}, \text{-}\pi_{j-1}, \ldots  \end{array} $$

Based on Eqs. 3, 4, 5, 6, 7 and 8, we developed a program in the GRSR tool to find and keep an record of independent transposition and block interchange events from *s* to *d* and search for repeats at the breakpoints of independent transposition and block interchange. The GRSR tool will then remove blocks involved in independent transposition and block interchange events in *s* and *d* before sorting by inversions using GRIMM. For example, in transforming the *permutation s* into *permutation d* in Eq. 2, Block 4 is found to be involved in an independent inverted transposition event using our GRSR tool. Since Block 4 substitutes Block 4, 5, and 6 in Eq. 1 in Step 1, the GRSR will eventually output Block 4, 5 and 6 are involved in a inverted transposition event. After writing this transposition event into the output Text file, GRSR will remove Block 4 in *permutation s* and *d* in Eq. 2, so *s* and *d* will become:

9 $$\begin{array}{*{20}l} s &= 1, 7, 8,9 \\ d &= 1, 7, 8,-9 \end{array} $$

For the purpose of using GRIMM in the next steps, GRSR make the permutation of *s* as 1, 2, 3,..., n and change the permutation of *d* accordingly. Hence *permutation s* and *d* in Eq. 9 will become:

10 $$\begin{array}{*{20}l} s &= 1, 2, 3, 4 \\ d &= 1, 2, 3, -4 \end{array} $$

In Eq. 10, we use Block 2, 3 and 4 to replace Block 7, 8 and 9 in Eq. 9, respectively. *Permutation s* and *d* like those in Eq. 10 will be the input of next step.

**Step 3: sorting by reversals using GRIMM.**

After eliminating blocks involved in independent transpositions and block interchanges, sorting by reversals will be conducted by using GRIMM. For the *permutation s* and *d* in Eq. 10, GRIMM will report a reversal of Block 4. As Block 4 replace the Synteny Block 9 in Step 2, GRSR will report Block 9 is involved in a reversal event.

Eventually, for the *permutation s* and *d* in Eq. 1, the GRSR will generate a rearrangement scenario as follows:

- Step 1: Block 4-6, inverted transposition.
- Step 2: Block 9, reversal.

Once a rearrangement event is found, the GRSR tool will compare the sequences at the breakpoints using BLAST [16] to see if there exist repeats which mediat the rearrangement event and write the result into the *report.txt* file. Our tool only searches for repeats which can make the sequences at the breakpoints remain unchanged before and after the rearrangement event. Hence, different rearrangement operations are mediated by repeats in different patterns:

*Pattern 1:* An *inversion* on region *π*_*i*_…*π*_*j*_ from *permutation s* to *d* is mediated by a pair of inverted repeats (A/-A), wheres = $\pi _{1},\ldots,\underbrace {A,\pi _{i},\pi _{i+1},\ldots,\pi _{j-1},\pi _{j},\text {-}A},\ldots,\pi _{n}$ andd = $\pi _{1},\ldots,\underbrace {A,\text {-}\pi _{j},\text {-}\pi _{j-1},\ldots,\text {-}\pi _{i+1},\text {-}\pi _{i},\text {-}A},\ldots,\pi _{n}$.

*Pattern 2:* An *independent transposition* on *π*_*i*_ from *permutation s* to *d* are mediated by three copies of directed repeats (A), where s = $\ldots,\pi _{i-1}, A,\underline {\pi _{i}},A, \underbrace {\pi _{i+1}, \ldots, \pi _{j-1}},A, \pi _{j}, \ldots $ andd = $\ldots,\pi _{i-1}, A,\underbrace {\pi _{i+1},\ldots,\pi _{j-1}},A,\underline {\pi _{i}},A,\pi _{j},\ldots $.

*Pattern 3:* An *independent inverted transposition* on *π*_*i*_ from *permutation s* to *d* are mediated by three copies of repeats (A), where s = $\ldots,\pi _{i-1}, A,\underline {\pi _{i}},A, \underbrace {\pi _{i+1}, \ldots, \pi _{j-1}},\text {-}A, \pi _{j}, \ldots $ andd = $\ldots,\pi _{i-1}, A,\underbrace {\pi _{i+1}, \ldots, \pi _{j-1}},\text {-}A, \underline {\text {-}\pi _{i}},\text {-}A, \pi _{j}, \ldots $.

*Pattern 4:* An *independent block interchange* on *π*_*i*_ and *π*_*j*_ from *permutation s* to *d* are mediated by two pairs of directed repeats (A and B), wheres = $\ldots,\pi _{i-1}, A,\underline {\pi _{i}},B, \pi _{i+1}, \ldots, \pi _{j-1}, A, \underline {\pi _{j}}, B, \pi _{j+1}, \ldots $ andd = $\ldots,\pi _{i-1}, A,\underline {\pi _{j}},B, \pi _{i+1}, \ldots, \pi _{j-1}, A, \underline {\pi _{i}}, B, \pi _{j+1}, \ldots $.

*Pattern 5:* An *independent inverted block interchange* on *π*_*i*_ and *π*_*j*_ from *permutation s* to *d* are mediated by two pairs of inverted repeats (A/-A, B/-B), wheres=$\ldots,\pi _{i-1}, A,\underline {\pi _{i}},B, \pi _{i+1}, \ldots, \pi _{j-1}, \text {-}B, \underline {\pi _{j}}, \text {-}A, \pi _{j+1}, \ldots $ andd=$\ldots,\pi _{i-1}, A,\underline {\text {-}\pi _{j}},B, \pi _{i+1}, \ldots, \pi _{j-1}, \text {-}B, \underline {\text {-}\pi _{i}}, \text {-}A, \pi _{j+1}\ldots $.

*Definition 6:* A *half inverted block interchange* on *π*_*i*_ and *π*_*j*_ from *permutation s* to *d* are mediated by four copies of repeat (A), wheres=$\ldots,\pi _{i-1}, A,\underline {\pi _{i}},\text {-}A, \pi _{i+1}, \ldots, \pi _{j-1}, A, \underline {\pi _{j}}, \text {-}A, \pi _{j+1}, \ldots $ intod$=\ldots,\pi _{i-1}, A,\underline {\pi _{j}},\text {-}A, \pi _{i+1}, \ldots, \pi _{j-1}, A, \underline {\text {-}\pi _{i}}, \text {-}A, \pi _{j+1}\ldots $.

## Results

We applied the GRSR tool on complete genomes of 28 *Mycobacterium tuberculosis* strains and 24 *Shewanella* strains. The respective results are described in the “Mycobacterium tuberculosis” and “Shewanella” sections.

### Mycobacterium tuberculosis

The complete genomes of these 28 strains were downloaded from NCBI GenBank (https://www.ncbi.nlm.nih.gov/genbank/). In this paper, each of the 28 strains is represented by a distinct number from 1 to 28. Strain 1 to 28 stands for *Mycobacterium tuberculosis* strain ZMC13-88, BT1, CCDC5079, F11, BT2, CCDC5180, Kurono, KZN 4207, K, EAI5, H37Rv (NC_018143), H37Rv (NC_000962), Haarlem, KIT87190, CTRI-2, 7199-99, KZN 1435, 96075, 49-02, KZN 605, Erdman (ATCC35801), CCDC5180, CDC1551, EAI5/NITR206, H37Ra; ATCC 25177, HKBS1, ZMC13-264, Beijing/NITR203, respectively. There are a total of 122.9 Mbp for these 28 genomes.

The Mugsy tool completed the multiple sequence alignment of these 28 unichromosomal genomes in <2 days and the alignment result was saved in an MAF file. The coordinates of core blocks were then extracted and written into the *core_coords.txt* file.

By utilizing the coordinates of core blocks, GRIMM-Synteny constructed 21 synteny blocks and generated 28 permutations for each input genomes, which took less than 2 minutes. The 28 permutations are listed in Table 1. The coordinates of the synteny blocks on each input genomes were saved in the *blocks.txt* file and the permutations were written in the *mgr_macro.txt* file.

**Table 1:** Permutations for Strain 1 to 28

Permutation
1 2 3 4 5 6 7 8 9 10 11 12 13 14 15 16 17 18 19 20 21
1 2 3 4 5 6 7 8 9 11 10 12 13 14 15 16 17 18 19 20 21
1 2 3 4 5 6 7 8 9 10 11 12 13 14 15 16 17 18 19 20 21
1 2 3 4 5 6 7 8 9 10 11 12 13 14 15 16 17 18 19 20 21
1 2 3 4 5 6-21 7 8 9 10 11 12 14 15-13 16 17 18 19 20
1 2 3 4 5 6 7 8 9 10 11 12 13 14 15 16 17 18 19 20 21
1 2 3 4 5 6 7 8 9 10 11 12 13 14 15 16 17 18 19 20 21
1 2 3 4 5-14-13-12-11-10-9-8-7-6 15 16 17 18 19 20 21
1 2 3 4 5 6 7 8 9 10 11 12 13 14 15 16 17 18 19 20 21
1 2 3 4 5 6 7 8 9 10 11 12 13 14 15 16 17 18 19 20 21
1 2 3 4 5 6 7 8 9 10 11 12 13 14 15 16 17 18 19 20 21
1 2 3 4 5 6 7 8 9 10 11 12 13 14 15 16 17 18 19 20 21
1 2 3 4 5 6 7 8 9 10 11 12 13 14 15 16 17 18 19 20 21
1 3 4 5 6 7 -8 9 10 11 12 13 14 15 16 2 17 19 18 20 21
1 2 3 4 5 6 7 8 9 10 11 12 13 14 15 16 17 18 19 20 21
1 2 3 4 5 6 7 8 9 10 11 12 13 14 15 16 17 18 19 20 21
1 2 3 4 5-14-13-12-11-10-9-8-7-6 15 16 17 18 19 20 21
1 2 3 4 5 6 7 8 9 10 11 12 13 14 15 16 17 18 19 20 21
1 2 3 4 5 6 7 8 9 10 11 12 13 14 15 16 17 18 19 20 21
1 2 3 4 5-14-13-12-11-10-9-8-7-6 15 16 17 18 19 20 21
1 2 3 5 6 7 8 9 10 11 12 13 14 15 4 16 17 18 19 20 21
1 2 3 4 5 6 7 8 9 10 11 12 13 14 15 16 17 18 19 20 21
1 2 3 4 5 6 7 8 9 10 11 12 13 14 15 16 17 18 19 20 21
1 2 3 4 5 6 7 8 9 10 11 12 13 14 15 16 17 18 19 20 21
1 2 3 4 5 6 7 8 9 10 11 12 13 14 15 16 17 18 19 20 21
1 2 3 4 5 6 7 8 9 10 11 12 13 14 15 16 17 18 19 20 21
1 2 3 4 5 6 7 8 9 10 11 12 13 14 15 16 17 18 19 20 21
1 2 3 4 5 6 7 8 9 10 11 12 13 14 15 16 17 18 19 20 21

Next, The genome rearrangement scenarios of 378 pairs of genomes were calculated in less than 10 minutes (there are 378 distinct pairs for 28 input genomes). And for each rearrangement event, the GRSR tool reported whether there were repeats flanking the rearranged regions. Here is a rearrangement scenario generated by the GRSR tool:

- Strain 1 to 8: total 1 rearrangement step(s):
  Step 1: Block 6-14, reversal. (Strain 1: Not found. Strain 8: Found, Length = 1355, Similarity = 100%)

According to the above results, we can see from Strain 1 to 8, there is a total of 1 rearrangement step which is a reversal of Block 6-14 and in Strain 8, the reversal region (Block 6-14) is flanked by a pair of inverted repeats with length = 1355 bp and similarity = 100%.

**Fig. 1:**
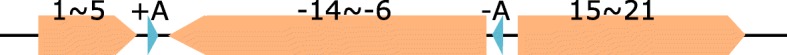
Permutation for Strain 8, 17 and 20. Each orange block stands for one or several consecutive synteny blocks. The integers above each orange block indicate the included synteny blocks, for example, -14$\thicksim $-6 means the orange block includes the synteny blocks from Block -14 to -6 on the permutation. Inverted repeat +A and -A is represented by triangles respectively. The arrow directions indicate the sign of each block

From the result generated by GRSR, we found that in Strain 8, 17, 20, the reversal region 6-14 are flanked by a pair of inverted repeats with length = 1355 bp and similarity = 100% as shown in Fig. 1. We can find that this pair of inverted repeats +A and -A can make sure the ends of the reversal region remain unchanged before and after the reversal event.

### Shewanella

There are totally 24 strains with complete genomes for the *Shewanella* Genus in NCBI GenBank when we conducted this experiment. We downloaded all these 24 *Shewanella* strains and applied our GRSR tool on these 24 strains. There is a total 121 Mbp for these 24 input genomes.

**Fig. 2:**
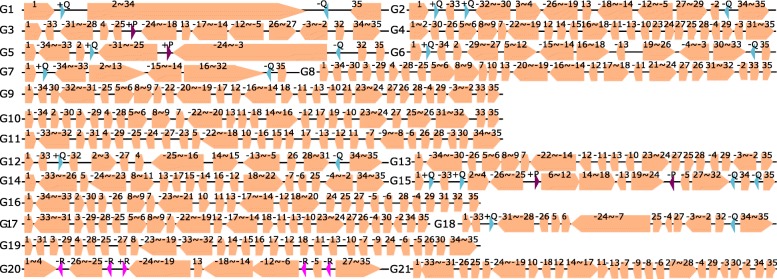
Twenty one kinds of permutations for the 24 *Shewanella* strains. Repeats P, Q and R are represented by triangles respectively. The arrow directions indicate the sign of each block

The GRSR tool achieved 35 synteny blocks for these 24 *Shewanella* genomes. The 24 genomes are divided into 21 groups according to their permutations. Genomes of the same permutation are in the same group. The permutations of the 21 groups (denoted as G1 to G21) are shown in Fig. 2. Group 6 consists of two *Shewanella baltica* strains OS155 and OS117. Group 15 includes three strains which are *Shewanella baltica* strains OS678, OS195 and OS185. Each of the remaining groups only has one strain: Group 1 to Group 5 corresponds to *Shewanella putrefaciens* CN-32, *Shewanella baltica* BA175, *Shewanella* sp. ANA-3, *Shewanella halifaxensis* HAW-EB4, *Shewanella* sp. W3 18-1 respectively; Group 7 to Group 14 contains *Shewanella putrefaciens* 200, *Shewanella violacea* DSS12, *Shewanella woodyi* ATCC 51908, *Shewanella sediminis* HAW-EB3, *Shewanella denitrificans* OS217, *Shewanella* sp. MR-7, *Shewanella pealeana* ATCC 700345, *Shewanella oneidensis* MR-1 respectively; Group 16 to Group 20 contains *Shewanella amazonensis* SB2B,*Shewanella loihica* PV-4, *Shewanella* sp. MR-4, *Shewanella piezotolerans* WP3, and *Shewanella baltica* OS223 respectively.

Ten pairs of groups with reversal distance = 1 are found. As the chromosome of *Shewanella* is circular, the reversal region can contain the two ends of a permutation. For example, for reversal of Block 34$\thicksim $1 from Group 1 to 2, denoted as (34,1) in Table 2, the blocks involved in the reversal events are Block 34, 35 and 1. All the 10 reversals (with lengths 56404 bp to 3440716 bp) are flanked by a pair of IRs (as listed in Table 2). In the 10 reversals, we found a total of three pairs of inverted repeats +P/-P, +Q/-Q and +R/-R. The locations of these three inverted repeats on the permutations are shown in Fig. 2. Six reversals are flanked by +Q/-Q, two reversals are mediated by +P/-P, one reversal is associated with +R/-R and the remaining one reversal between Group 15 to Group 20 is special because the inverted segment (6,24) is flanked by +P/-P in strains of Group 15 but by +R/-R in strains of Group 20. Sequence P is about 195 bp length, Q is about 5597 bp and R is about 1218 bp. Their lengths vary slightly in different strains.

**Table 2:** Summary of the characteristics of reversals mediated by IR with reversal distance = 1

sG	dG	rev _*d*_	reversal	len (Mbp)	IR	r _*d*_
1	2*	1	(34,1)	0.1530	Q(1)	3
1	5	1	(3,31)	3.4407	Q(2)	2
1	7*	1	(35,1)	0.0834	Q(2)	2
1	20	1	(6,24)	2.2358	R(1)	3
2	15*	1	(34,-33)	0.3553	Q(2)	2
3	15	1	(-24,-6)	2.6924	P(1)	3
5	15	1	(-24,-6)	2.4606	P(1)	4
6	15*	1	(35,1)	0.0564	Q(2)	3
12	18*	1	(34,-33)	0.2948	Q(2)	2
15	20	1	(6,24)	2.7479	P(0)	2
					R(1)	

Column *sG* and *dG* are the source and destination group. Rearrangement scenario is calculated from the permutation of the source group to the destination group. For dG with a asterisk, the reversal event is calculated from the permutation of sG to permutation of dG in the negative strand. For example, if the permutation of dG is 1, 2, 3, then the permutation of dG in the negative strand is -3, -2, -1. Column *rev*_*d*_ indicates the reversal distance between sG and cG after eliminating other independent rearrangement events. Column *r*_*d*_ indicates the distance of independent rearrangement events other than reversals. *len* is the length (in mbp) of reversal. Column *IR* lists which pair of inverted repeats (P, Q or R) flanks the reversal. The numeric code: 0 indicates the respective IR was found only in the source group, 1 indicates the IR was found only in the closest group, 2 indicates the IR was found in both groups

**Fig. 3:**
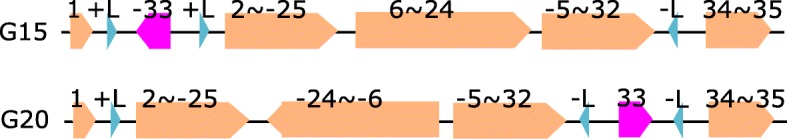
Inverted transposition of Block -33 from G15 to G20. +L and -L are repeats at the breakpoints of this transposition

We also find repeats at the breakpoints of inverted transpositions and inverted block interchanges in *Shewanella* strains. For example, from the permutation of G15 to that of G20, Block -33 is involved in an inverted transposition (See Fig. 3). The repeats denoted as +L/-L at the breakpoints of the transposition are also shown in Fig. 3. We can find that the three existence of repeat L can make sure the ends of the transposition remain unchanged before and after this inverted transposition.

**Fig. 4:**
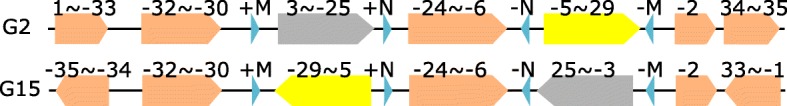
Inverted block interchange between the gray and yellow region. +M/-M and +N/-N are repeats at the breakpoints of this block interchange

Figure 4 shows repeats at the breakpoints of an inverted block interchange. From the permutation of G2 to G20, Block 3 to -25 (the gray region in Fig. 4) and Block -5 to 29 (the yellow region in Fig. 4) are involved in an inverted block interchange event. We can find that the existence of two pairs of inverted repeats +M/-M and +N/-N make the ends of the interchanged regions remain unchanged.

## Discussion

Lots of algorithms have been proposed for computing rearrangement events with different kinds of rearrangement events such as reversals, transpositions, block interchanges, etc. However, it is hard to estimate in what sense the computed results reflect the true evolutionary history due to multiple solutions (especially when a large number of rearrangement events are involved) and different kinds of models for incorporating different kinds of rearrangement events. The GRIMM tool sorts permutations by reversal events only. As one transposition or block interchange event can be replaced by 3 reversal events, and one inverted transposition or inverted block interchange event can be replaced as 2 reversal events, the calculated reversal steps by GRIMM may not be the real reversal events. As a result, to better study the association between repeats and rearrangement events, our tool is designed to identify solid and obvious (independent) rearrangement events before searching for repeats at the breakpoints of rearrangement events

For current methods, computing the rearrangement distance between two chromosomes also suffers from multiple solutions. Incorporating the mechanism that rearrangement events are associated with repeats will reduce the chance of multiple solutions.

In this paper, we only consider the three operations reversals, block interchanges and transpositions where only one chromosome is involved. Further study will include rearrangement operations such as translocations, fissions and fusions where two chromosomes are involved.

## Conclusions

From the results generated by the GRSR tool, we observed that many reversal events were flanked by a pair of inverted repeats so that the two ends of the reversal region remained unchanged before and after the reversal event. We also observed that in other rearrangement operations such transposition and block interchange, there existed repeats (not necessarily inverted) at the breakpoints, where the ends remained unchanged before and after the rearrangement operations. This suggests that the conservation of ends could possibly be a popular phenomenon in many types of genome rearrangement events.

## Abbreviations

IR: Inverted repeat
Mbp: Million base pair
